# Identification of Major Loci and Candidate Genes for Meat Production-Related Traits in Broilers

**DOI:** 10.3389/fgene.2021.645107

**Published:** 2021-03-30

**Authors:** Xinting Yang, Jiahong Sun, Guiping Zhao, Wei Li, Xiaodong Tan, Maiqing Zheng, Furong Feng, Dawei Liu, Jie Wen, Ranran Liu

**Affiliations:** ^1^State Key Laboratory of Animal Nutrition, Key Laboratory of Animal (Poultry) Genetics Breeding and Reproduction, Ministry of Agriculture, Institute of Animal Sciences, Chinese Academy of Agricultural Sciences, Beijing, China; ^2^Foshan Gaoming Xinguang Agricultural and Animal Industrials Corporation, Foshan, China

**Keywords:** carcass composition, thigh meat, weight trait, genome-wide association study, imputation, candidate genes

## Abstract

**Background:**

Carcass traits are crucial characteristics of broilers. However, the underlying genetic mechanisms are not well understood. In the current study, significant loci and major-effect candidate genes affecting nine carcass traits related to meat production were analyzed in 873 purebred broilers using an imputation-based genome-wide association study.

**Results:**

The heritability estimates of nine carcass traits, including carcass weight, thigh muscle weight, and thigh muscle percentage, were moderate to high and ranged from 0.21 to 0.39. Twelve genome-wide significant SNPs and 118 suggestively significant SNPs of 546,656 autosomal variants were associated with carcass traits. All SNPs for six weight traits (body weight at 42 days of age, carcass weight, eviscerated weight, whole thigh weight, thigh weight, and thigh muscle weight) were clustered around the 24.08 Kb region (GGA24: 5.73–5.75 Mb) and contained only one candidate gene (*DRD2*). The most significant SNP, rs15226023, accounted for 4.85–7.71% of the estimated genetic variance of the six weight traits. The remaining SNPs for carcass composition traits (whole thigh percentage and thigh percentage) were clustered around the 42.52 Kb region (GGA3: 53.03–53.08 Mb) and contained only one candidate gene (*ADGRG6*). The most significant SNP in this region, rs13571431, accounted for 11.89–13.56% of the estimated genetic variance of two carcass composition traits. Some degree of genetic differentiation in *ADGRG6* between large and small breeds was observed.

**Conclusion:**

We identified one 24.08 Kb region for weight traits and one 42.52 Kb region for thigh-related carcass traits. *DRD2* was the major-effect candidate gene for weight traits, and *ADGRG6* was the major-effect candidate gene for carcass composition traits. Our results supply essential information for causative mutation identification of carcass traits in broilers.

## Introduction

For global meat consumption, chicken meat is the second largest and provide almost 1/3 of meat resource^1^. Improvements in the weight and carcass traits are major goals in modern broiler breeding programs. In particular, thigh development and meat production are closely related to the efficiency of the broiler industry. These traits have moderate to high heritability and are controlled by multiple genes (Claire D’Andre et al., 2013; Flisar et al., 2014).

Many studies have been performed to identify quantitative trait loci (QTLs), genes, and/or causative mutation. In pigs, a causative mutation of *IGF2* has a major effect on muscle growth (Van Laere et al., 2003). In large dog breeds, variants in *IRS4*, *ACSL4*, and *IGSF1* were strongly associated with skeletal size and body mass (Plassais et al., 2017). In mice and humans, changes in the Neurobeachin abundance or activity significantly affect the body weight (Olszewski et al., 2012). A non-synonymous *FGD3* variant was identified as a positional candidate for disproportional tall stature, accounting for a carcass weight QTL and skeletal dysplasia in Japanese Black cattle (Takasuga et al., 2015). Many QTL and genes have been found in chickens, including *LCORL1* and *LDB2* (Gu et al., 2011; Liu et al., 2013, 2015). Recently, multiple haplotypes at the distal end of chromosome 1 were identified as a major-effect QTL for chicken growth traits (Wang et al., 2020). However, the genetic mechanisms of carcass traits are not well understood.

An imputation-based genome-wide association study was conducted to identify significant loci and candidate genes affecting multiple weight and carcass composition traits in fast-growing white-feathered broilers.

## Materials and Methods

### Experimental Birds

Chickens were obtained from a fast-growing white-feathered broiler line B. The chickens were produced and raised by Foshan Gaoming Xinguang Agricultural and Animal Industrials Co., Ltd. (Foshan, China). In generation 5 (G5), 873 breeders (412 males and 462 females) were randomly selected and slaughtered at 42 day of age. All birds were raised in stair-step cages under the same recommended environmental (Li et al., 2009) and nutritional conditions (Feeding Standard of Chickens, China, NY 33-2004).

In addition, thirty Chahua and 24 Daweishan mini chickens were used for the phylogenetic and selective sweep analysis. Chahua chickens are similar to red junglefowl. The blood samples were supplied by the Aquatic Animal Husbandry Association of Yulin City in the Guangxi Zhuang Autonomous Region. The individuals are small, and the body weight rarely exceeds 1 kg. The blood samples of the Daweishan mini chickens were obtained from the Yunnan Agricultural University. The Daweishan mini chickens are a small-sized Chinese indigenous breed similar to junglefowl distributing in the tropical and subtropical zone in Yunnan Province. The typical characteristics of Daweishan Mini chickens are a slow growth rate and low body weight compared with Chinese indigenous chickens of the same age (Rong et al., 2011).

### Phenotypic Measurements

The weight traits and carcass composition traits were measured in line B as follows: body weight at 42 day of age (BW42), carcass weight (CW), eviscerated weight (EW), whole thigh weight (WThW, weight including feet, and single), thigh weight (ThW, weight with bone, and single), thigh muscle weight (ThMW, single), and the WThW, ThW, and ThMW as percentages of BW42 (WThP, ThP, and ThMP). In addition, the same method (Li D. et al., 2020) was used to calculate the feed intake from 28 to 42 days of age (FI).

### Estimates of Genetic Parameters

A linear mixed model (LMM) was fitted using the residual maximum likelihood (REML). The ASReml software (Gilmour et al., 2009) was used to estimate the variance components of the carcass traits of 873 broilers. The Wald *F*-statistics showed that the fixed effect of sex was statistically significant (*P* < 0.05). The variance components were estimated using a univariate mixed liner animal model. The animal model was expressed as follows:

Y=X⁢b+Z⁢u+e

where *Y* is the vector of observations; *b* is the vector of the fixed effects; *u* is the vector of the additive genetic effects, with u $∼N\,\left(0,A\,\sigma_{u}^{2}\right)$, where *A* is the additive genetic relationship matrix (GRM), and $\sigma_{u}^{2}$ is the additive genetic variance; *e* is the vector of the random residual effects; and *X* and *Z* are the incidence matrices assigning observations to effects. The fixed effect in the model was sex.

The phenotypic variance is the sum of all variance components and is defined as follows:

$$\sigma_{P}^{2}=\sigma_{a}^{2}+\sigma_{e}^{2}$$

The heritability is the ratio of the additive genetic variance to the phenotypic variance and is defined as follows:

$$h^{2}=\frac{\sigma_{a}^{2}}{\sigma_{P}^{2}}$$

where $\sigma_{P}^{2}$ is the phenotypic variance, $\sigma_{a}^{2}$ is the additive variance, and $\sigma_{e}^{2}$ is the residual fraction.

A bivariate animal model was fitted to estimate the phenotypic and genetic correlations between carcass traits using the ASReml software package. The description of the model terms was the same as that for the univariate mixed linear animal model.

A likelihood ratio test (LRT) was used to determine the significance of the heritability and the genetic correlations. The LRT compares the likelihood of a full model with that of a nested model (without the additive genetic component) and was used to test for nonzero additive genetic variance.

### Genotyping, Imputation, and Quality Control

Genomic DNA was extracted from blood samples using the phenol-chloroform method. The 873 broilers were genotyped with the customized chicken 55 K SNP array obtained from Beijing Compass Biotechnology Co., Ltd. (Beijing, China; Liu et al., 2019). A total of 873 broilers (412 males and 462 females) were used for genotype imputation for the target panel. The following quality control criteria were used for the target panel: individual call percentage ≥90%, SNP call percentage ≥90%, and minor allele frequency (MAF) ≥0.05. In addition, the SNPs located on the sex chromosomes and GGA16 were removed. We used 41,204 autosome variants and 873 broilers in the subsequent analyses.

Whole-genome sequences (WGS) of 230 broilers (101 males and 129 females) from the same line in G7 were used for the reference panel (Li D. et al., 2020). Briefly, an average depth of 10× was acquired, and variant calling and SNP filtering were performed according to a standardized bioinformatics pipeline. The quality control criteria of the reference panel included the individual call percentage ≥90%, SNP call percentage ≥90%, and MAF ≥ 0.05. After filtering, 9,760,228 autosome variants remained for 230 birds.

For the Chahua and Daweishan mini chickens, genome resequencing was conducted on the Illumina NovaSeq 6000 platform with an average depth of approximately 10× coverage. The sequencing was performed by Annoroad Gene Technology Co., Ltd. (Beijing, China). Variant calling was performed according to a standardized bioinformatics pipeline (DePristo et al., 2011; Van der Auwera et al., 2013). Specifically, clean sequencing data were aligned to the chicken reference genome (GRCg6a/galGal6; ftp://ftp.ncbi.nlm.nih.gov/genomes/all/GCF/000/002/315/GCF_000002315.6_GRCg6a/) with the Burrows-Wheeler Aligner (BWA)-MEM algorithm (Li, 2013). Then, PCR duplicates were removed with Picard-tools v1.115^2^. Variant calling was then performed via the HaplotypeCaller in GVCF mode with joint genotyping on all samples. We used ANNOVAR software (Wang et al., 2010) and the existing genome annotation file (gff) to annotate each detected SNP. Finally, the SNPs were filtered with the GATK Variant Filtration protocol. The filtering settings were as follows: variant confidence score < 30.0, QualByDepth < 2.0, ReadPosRankSum < −8.0, total depth of coverage < 4.0, and FisherStrand > 60.0. In addition, quality control was conducted using the following criteria: individual call rate ≥ 90%, SNP call rate ≥ 90%, and MAF ≥ 0.05. After filtering, a total of 9,235,705 autosome variants remained for the 54 sequenced birds.

Genotype imputation of the 55 K genotypes of the broilers to the imputed WGS level was performed with Beagle 5.0 (Browning et al., 2018). The effective population size (Ne) affects the accuracy of genotype imputation (Van den Berg et al., 2019) because it is much smaller in livestock than in humans (Hall, 2016). We used SNeP software (Barbato et al., 2015) to estimate the Ne; SNeP estimates the Ne using the following equation:

$$N_{T}\,\left(t\right)=\frac{1}{4\,f\,\left(c_{t}\right)}\times \left[\frac{1}{E\,\left(r_{a\,d\,j}^{2}\,v\,c_{t}\right)}-a\right]$$

where *N*_*T*_(*t*) is the effective population size estimated *t* generations ago, *f*(*c*_*t*_) is the mapping function used to estimate the recombination rate (*c*_*t*_) *t* generations ago, $r_{a\,d\,j}^{2}$ is the linkage disequilibrium (LD) estimate adjusted for the sampling bias ($r_{a\,d\,j}^{2}=r^{2}-\frac{1}{2\,N}$, where *N* is the population sample size), and “*a*” is a constant accounting for mutation. The results showed that the Ne was 432 before the 13th generation.

The reference panel was pre-phased with Beagle 5.0 (default settings, except that the Ne was 432; Browning et al., 2018). Then the imputation from 55 K to the WGS level was executed in Beagle 5.0 with the default parameters, except that the Ne was 432. To assess the imputation accuracy, the allelic *R*^2^ values for each variant and the genotype concordance rate for a random 2% SNPs were determined. The allelic *R*^2^ was calculated as the estimated squared correlation of the imputed sequence genotype on the true sequence, which was given by Beagle 5.0. The genotype concordance rate was calculated by comparing the imputed and real genotypes for a randomly masked 2% of the SNPs analyzed with both panels. We applied the following post-imputation filtering criteria for each SNP: allelic *R*^2^ ≥ 0.8 and MAF ≥ 0.05. Finally, 6,546,656 autosomal variants and 873 samples remained for the subsequent genome−wide association study (GWAS; Supplementary Table 1).

### Genome-Wide Association Study

The GWAS was performed using the univariate LMM implemented in GEMMA version 0.98.1 software^3^ (Zhou and Stephens, 2012). Due to the high genetic correlation between the weight traits and carcass composition traits, we fitted a multivariate linear mixed model (MLMM) for the weight traits and carcass composition traits. The genotype was the fixed effect, and the additive polygenic effect was the random effect. Sex was considered a covariate for all traits.

The univariate LMM had the following form:

$$y=W\,\alpha +x\,\beta +u+\varepsilon ;u∼M\,V\,N_{n}\,\left(0,\lambda \,\tau^{-1}\,K\right),\;\,\varepsilon ∼M\,V\,N_{n}\,\left(0,\tau^{-1}\,I_{n}\right),$$

where *y* represents the vector of the phenotypic values; *W* represents the vector of the covariates, including a column of 1 s; α represents the vector of the corresponding coefficients, including the intercept; *x* represents the vector of the marker genotypes; β represents the effect size of the marker; *u* represents the vector of the random polygenic effects; ϵ represents the vector of errors; τ^−1^ represents the variance of the residual errors; λ represents the ratio between the two variance components; *K* represents the centered relatedness matrix estimated from 6,546,656 variants, and In represents the identity matrix. *MVN*_*n*_ denotes the *n*-dimensional multivariate normal distribution. The Wald test was used as a criterion to select the SNPs associated with the carcass traits.

The MLMM had the following form:

$$Y=W\,A+x\,\beta^{T}+U+E;G∼M\,N_{n\times d}\,\left(0,K,V_{g}\right),\;\,E∼M\,N_{n\times d}\,\left(0,I_{n\times n},V_{e}\right),$$

where *Y* is an *n* × *d* matrix of *d* phenotypes for *n* individuals; *W* = (*w*_1_, ⋅⋅⋅, *w*_*c*_) is an *n* × *c* matrix of covariates, including a column of 1 s; *A* is a *c* × *d* matrix of the corresponding coefficients, including the intercept; *x* is an *n*-vector of the marker genotypes; β is a *d* vector of the marker effect sizes for the *d* phenotypes; *U* is an *n* × *d* matrix of the random effects; *E* is an *n* × *d* matrix of the errors; *K* is the centered relatedness matrix estimated from 6,546,656 variants, *I*_*n  ×n*_ is a *n* × *n* identity matrix, *V*_*g*_ is a *d* × *d* symmetric matrix of the genetic variance components, *V*_*e*_ is a *d* × *d* symmetric matrix of the environmental variance components, and *MN*_*n×d*_ (0, *V*_1_, *V*_2_) denotes the normal distribution of the *n* × *d* matrix with mean 0, a row covariance matrix *V*_1_ (*n* by *n*), and a column covariance matrix *V*_2_ (*d* by *d*). The Wald test was used as a criterion to select the SNPs associated with the carcass traits.

The threshold *P*-value of the 5% Bonferroni genome-wide significance was 7.63e-9 (0.05/6,546,656), and that of the significance of the “suggestive association” that allows a one-time false positive effect in the GWAS test was 1.52e-7 (1/6,546,656). It was calculated using the same method. Manhattan and quantile-quantile (Q-Q) plots were constructed for each trait using the CMplot package^4^ in R (version 4.0.0). LD blocks of the target regions were identified using the Haploview version 4.2 software (Barrett et al., 2005). The SNP positions were updated using the newest release from the University of California-Santa Cruz (UCSC; GRCg6a/galGal6 genome version). The identification of genes in the genome-wide significant and suggestive regions was performed using the UCSC annotation of the GRCg6a/galGal6 genome version^5^. Boxplots were produced with the ggplot2 package in R (version 4.0.0).

### Bayesian Analysis

A Bayesian approach called Bayes *C*_π_ (Habier et al., 2011) was used to obtain the average proportion of genetic variance and phenotypic variance explained by each 1-Mb genomic non-overlapping window (*n* = 1,024). The Bayesian analyses were performed using the hibayes package^6^ in R (version 4.0.0). The number of iterations after the burn-in phase was 20,000, and that of the burn-in period was 10,000. Sex was fitted as a fixed covariate for all traits.

### Estimation of SNP Effect Size

The estimation of SNP effect size was performed using MLMM analysis with the genotype data used to compute the GRM in DISSECT (Canela-Xandri et al., 2015)^7^. The approach is based on fitting the equation:

y=X⁢β+W⁢u+ϵ

where *y* is the vector of the phenotypes, β is the vector of fixed effects that included sex, *u* is the vector of the SNP effects distributed as $u∼N\,\left(0,I\,\sigma_{u}^{2}\right)$, *I* is the identity matrix, and ϵ is the vector of the residual effects distributed as ∼*N*(0,*I*σ^2^). W is a genotype matrix defined by the equation:

$$w_{i\,k}=\frac{\left(s_{i\,k}-2\,p_{k}\right)}{\sqrt{2\,p_{k}\,\left(1-p_{k}\right)}}$$

where *s*_*ik*_ is the number of copies of the reference allele for the SNP *k* of the individual *i*, and *p*_*k*_ is the frequency of the reference allele for the SNP *k*. In this model, the variance of *y* is:

$$\text{var}\,\left(y\right)=A\,\sigma_{g}^{2}+I\,\sigma^{2}$$

where A is the GRM, $\sigma_{g}^{2}$ is the genetic variance, and σ^2^ is the residual variance. The variance components were estimated using the REML.

### Phylogenetic Analysis

Using data from 80 white-feathered broilers with different-parents, 30 Chahua chickens, and 24 Daweishan mini chickens, the pair-wise genetic distance matrices were calculated based on whole-genome SNPs, two candidate genes, and five randomly selected 100 Kb regions, respectively. A total of eight neighbor-joining trees were then constructed using MEGA X (Kumar et al., 2018). The defaults were used for all parameters.

### Selective Sweep Analysis

Selective sweep analysis was performed on 80 white-feathered broilers with different parents, 30 Chahua chickens, and 24 Daweishan mini chickens. The population differentiation index (*F*_*ST*_) was calculated as described by Weir and Cockerham (1984) and was implemented in the VCFtools v0.1.14 program (Danecek et al., 2011). The average *F*_*ST*_ values were plotted in 20-kb overlapping genomic bins (for more than 10 SNPs) with a 10-kb step-size.

The nucleotide diversity (π) of each population was estimated using a 20-kb sliding window (for more than 10 SNPs) with a 10-kb step across the whole genome, and the ratio (π_*ChahuaandMINI*_/π_B*line*_) was computed. The *log*_2_⁡(πratio) was defined as $\log_{2}\frac{\pi_{\mathrm{Chahua}\,\mathrm{and}\,\mathrm{MINI}}}{\pi_{\mathrm{line}\,B}}$.

The heterozygosity *H*_*P*_ was calculated only in the line B chickens as: $H_{P}=\frac{2\,\sum n_{M\,A\,J}\,\sum n_{M\,I\,N}}{{\left(\sum n_{M\,A\,J}+\sum n_{M\,I\,N}\right)}^{2}}$, where ∑*n*_*MAJ*_ is the sum of the major allele frequencies, and ∑*n*_*MIN*_ is the sum of the minor allele frequencies within a window. The *H*_*P*_ value was Z-transformed as follows: $Z\,H_{P}=\frac{\left(H_{P}-\mu \,H_{P}\right)}{\sigma \,H_{P}}$, where μ*H*_*P*_ is the overall average heterozygosity and σ*H*_*P*_ is the standard deviation for all windows.

The cross-population extended haplotype homozygosity (XP-EHH; Sabeti et al., 2007) was calculated for each SNP in the dataset using Selscan v-1.3.0 (Szpiech and Hernandez, 2014). Prior to the XP-EHH test, Beagle 5.0 software (Browning et al., 2018) was used to estimate missing genotypes and reconstructing the haplotypes from the unphased SNP genotype data.

The overall experimental workflow is depicted in Supplementary Figure 1.

## Results

### Descriptive Statistics of Phenotypes

The descriptive statistics of the carcass traits are listed in Table 1. At 42 days of age, the body weight (BW42), carcass weight (CW), and eviscerated weight (EW) were 1912.74 ± 196.49, 1728.24 ± 180.26, and 1229.60 ± 145.98. The whole thigh weight (WThW, weight including feet), thigh weight (ThW, weight with bone), and thigh muscle weight (ThMW) were 249.47 ± 31.13, 213.37 ± 26.65, and 157.19 ± 20.20. The whole thigh percentage (WThP), thigh percentage (ThP), and thigh muscle percentage (ThMP) were 13.03 ± 0.74, 11.15 ± 0.67, and 8.21 ± 0.54. Feed intake from 28 to 42 days of age was 1775.98 ± 147.83. The coefficients of variation of these traits in the population ranged from 5.64 to 12.85%.

**TABLE 1:** Descriptive statistics of the carcass traits of broilers.

**Traits^1^**	***N***	**Mean**	**SD^2^**	**Min**	**Max**	**CV, %^3^**
BW42, g	873	1912.74	196.49	1327.60	2502.00	10.27
CW, g	873	1728.24	180.26	1168.90	2278.60	10.43
EW, g	873	1229.60	145.98	786.80	1724.60	11.87
WThW, g	873	249.47	31.13	164.86	343.06	12.48
ThW, g	873	213.37	26.65	135.80	293.20	12.49
ThMW, g	873	157.19	20.20	99.50	224.50	12.85
WThP, %	873	13.03	0.74	10.80	15.33	5.64
ThP, %	873	11.15	0.67	9.25	13.10	5.98
ThMP, %	873	8.21	0.54	6.46	11.36	6.63
FI, g	873	1775.98	147.83	1235.20	2130.20	8.32

*^1^Body weight at 42 days of age (BW42), carcass weight (CW), eviscerated weight (EW), whole thigh weight (WThW), thigh weight (ThW), thigh muscle weight (ThMW), whole thigh percentage (WThP), thigh percentage (ThP), thigh muscle percentage (ThMP), and feed intake from 28 to 42 days of age (FI). ^2^Standard deviation. ^3^Coefficient of variation (%).*

### Estimates of Genetic Parameters

The heritabilities and the phenotypic and genetic correlations for the carcass traits are presented in Table 2. The WThP and ThP had moderate heritabilities (0.21–0.22). The heritabilities of the other carcass traits were higher (0.30 to 0.39). All weight traits showed strong positive genetic correlations (0.78–0.99) and phenotypic correlations (0.71–0.99). All carcass composition traits showed strong positive genetic correlations (0.76–0.94) and phenotypic correlations (0.81–0.98).

**TABLE 2:** Overview of the significant QTLs of univariate GWAS and multivariate GWAS associated with target traits.

**Traits^1^**	**GGA^2^**	**Base-pair region**		**nSNP^3^**	**Lead SNP**	**Alleles**	**MAF**	***P*-value**	**Candidate gene**	**PVE(%)^4^**	**GVE(%)^5^**	**Position**
		**Start**	**End**		**(rsname)**							
BW42	24	5658679	5835194	14	5741556	C/G	0.23	4.06E-09	DRD2	2.75	4.85	Intron 1
CW	24	5658679	5754835	13	5741556	C/G	0.23	3.97E-09	DRD2	2.79	5.00	Intron 1
EW	24	5632110	5835194	49	5741556	C/G	0.23	3.46E-10	DRD2	3.43	7.71	Intron 1
WThW	24	5741556	5874395	5	5741556	C/G	0.23	1.21E-08	DRD2	2.81	5.96	Intron 1
ThW	24	5741556	5744643	3	5741556	C/G	0.23	2.62E-08	DRD2	2.72	5.89	Intron 1
ThMW	24	5741556	5744643	2	5741556	C/G	0.23	4.94E-08	DRD2	2.52	4.95	Intron 1
WThP	3	53032570	53075086	65	53034833	T/C	0.35	2.40E-09	ADGRG6	3.55	11.89	Intron 2
ThP	3	53032570	53075086	68	53034833	T/C	0.35	4.61E-09	ADGRG6	3.43	13.56	Intron 2
BW42, CW, EW	24	5658679	5744643	4	5741556	C/G	0.23	1.15E-08	DRD2	/	/	Intron 1
BW42, CW, EW, WThW, ThW, ThMW	24	5741556	5741556	1	5741556	C/G	0.23	1.28E-07	DRD2	/	/	Intron 1
WThP, ThP, ThMP	3	53032570	53757085	58	53033387	C/T	0.33	8.63E-09	ADGRG6	/	/	Intron 2

*^1^Body weight at 42 days of age (BW42), carcass weight (CW), eviscerated weight (EW), whole thigh weight (WThW), thigh weight (ThW), thigh muscle weight (ThMW), whole thigh percentage (WThP), thigh percentage (ThP), thigh muscle percentage (ThMP). ^2^Gallus gallus chromosome. ^3^The number of significant SNPs in the interval. ^4^Variance in phenotype explained by the lead SNP. ^5^Genetic variance explained by the lead SNP.*

### Imputation Accuracy

**FIGURE 1:**
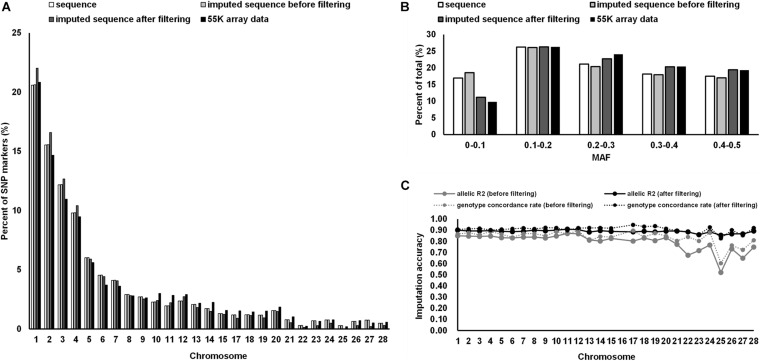
Distribution of SNP markers and imputation accuracy. **(A)** Percentage of SNPs on the chromosome for the 55 K array data of 873 birds, WGS data of 230 birds, and imputed WGS data of 873 birds after imputation and post-imputation filtering. **(B)** Percentage of SNPs in each MAF class for different datasets. **(C)** The imputation accuracy of the imputed WGS data of 873 birds after imputation and post-imputation filtering. MAF, minor allele frequency.

The proportion of SNP markers on each chromosome based on different datasets are shown in Figure 1A. The numbers of SNPs in the different MAF classes for different datasets are shown in Figure 1B. In general, the proportion of SNP markers on each chromosome and the MAF distribution of the four datasets showed the same trends. Consistency was observed in the distribution of SNPs between the 55 K array data and the imputed WGS data after filtering and between the WGS data and imputed WGS data before filtering (MAF ≥ 0.05).

The allelic *R*^2^ values and the average genotype concordance rate were used to evaluate the imputation accuracy of the imputed WGS data (Figure 1C). At the chromosome level, the allelic *R*^2^ values of the imputed sequence before filtering ranged from 0.52 to 0.87, whereas the genotype concordance rate fluctuated between 0.60 and 0.89. After post-imputation filtering, the allelic *R*^2^ values and the genotype concordance rate reached an average of 0.89 and 0.91, respectively. The distribution of the SNPs used in the GWAS after post-imputation filtering is summarized in Supplementary Table 1.

### GWAS Results

**FIGURE 2:**
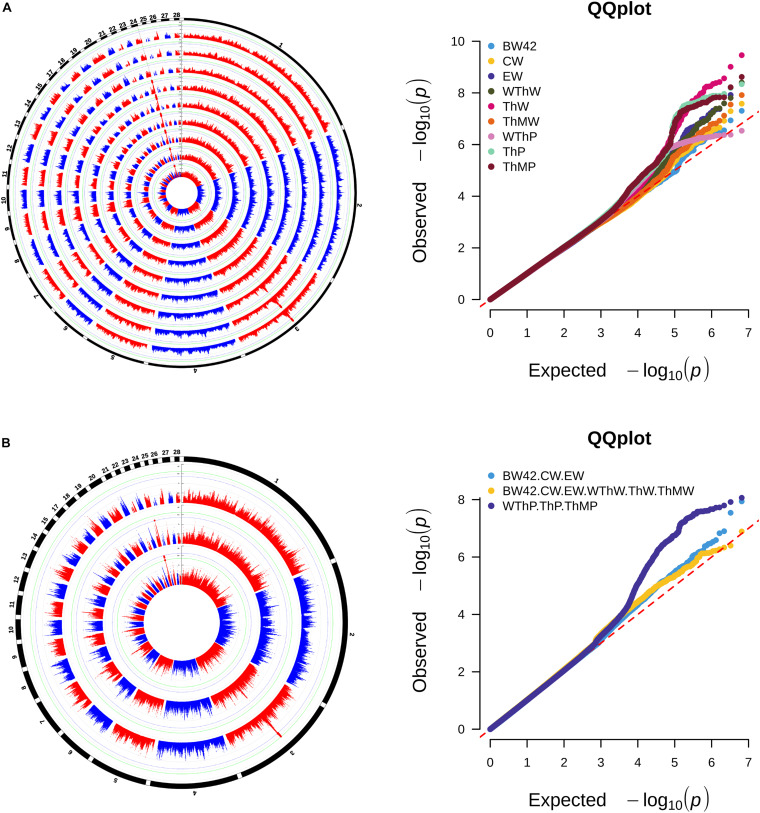
Manhattan and quantile-quantile plots of univariate GWAS **(A)** and multivariate GWAS **(B)** for carcass traits. Each dot represents an SNP in the dataset. The circle Manhattan plots from the inside to the outside are BW42, CW, EW, WThW, ThW, ThMW, WThP, ThP, and ThMP. The horizontal blue and green lines indicate the thresholds for genome-wide significance (*P*-value = 7.64e-9) and suggestive significance (*P*-value = 1.53e-7), respectively.

The Manhattan and Q-Q plots of the univariate GWAS results are presented in Figure 2A and Table 2. We detected 12 genome-wide significant SNPs and 118 suggestively significant SNPs. All SNPs for the weight traits (BW42, CW, EW, WThW, ThW, and ThMW) were clustered around the 242.29 Kb region (GGA24: 5.63–5.87 Mb). The most significant SNP, rs15226023, accounted for 4.85–7.71% of the genetic variance of the six weight traits. The remaining SNPs for the carcass composition traits (WThP and ThP) were clustered around the 42.52 Kb region (GGA3: 53.03–53.08 Mb). The most significant SNP in this region, rs13571431, accounted for 11.89 and 13.56% of the genetic variance of WThP and ThP, respectively.

The consistent significant loci were detected using the multivariate GWAS and univariate GWAS. The Manhattan and Q-Q plots of the multivariate GWAS are presented in Figure 2B and Table 2.

**FIGURE 3:**
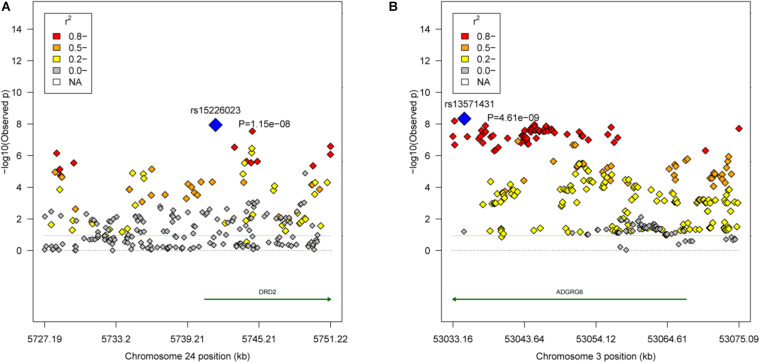
Regional association plots of the candidate areas. **(A)** The QTL region GGA24: 5.73–5.75 Mb associated with the weight traits. The blue dot represents the lead SNP rs15226023. **(B)** The QTL region GGA3: 53.03–53.08 Mb associated with the carcass composition traits. The blue dot represents the lead SNP rs13571431. Different levels of linkage disequilibrium (LD) between the lead SNP and the surrounding SNPs are shown in different colors (red: r^2^ ≥ 0.8; orange: 0.5 ≤ r^2^ < 0.8; yellow: 0.2 ≤ r^2^ < 0.5; and gray: r^2^ < 0.2). The gene annotations were obtained from the University of California Santa Cruz (UCSC) Genome Browser (http://genome.ucsc.edu).

Based on *r*^2^ ≥ 0.8, the empirical confidence interval of the QTL in the GGA24 for the weight traits was 24.08 kb (GGA24: 5.73–5.75 Mb), and the unique gene *DRD2* was located in the region (Figure 3A). Another QTL in the GGA3 for carcass composition traits was 42.52 kb (GGA3: 53.03–53.08 Mb), and the unique gene *ADGRG6* was located in the region (Figure 3B).

For the weight traits, the 24.83 Kb region in GGA24 (GGA24: 5.73–5.75 Mb) in the significant region was detected by LD analysis (Supplementary Figure 2A). This LD block covered the exon1 and intron1 of *DRD2* and had a positive effect (β < 0) on all weight traits (Supplementary Figure 2B). Interestingly, this LD block also had a positive effect (β < 0) on FI (Supplementary Figure 2B).

For WThP and ThP, one 42.52 kb strong LD block in GGA3 (GGA3: 53.03–53.08 Mb) was detected by LD analysis and contained 69 significant SNPs (Supplementary Figure 3A). This LD block covered exon1, intron1, exon2, and intron2 of *ADGRG6* and had a negative effect (β < 0) on all carcass composition traits (Supplementary Figure 3B).

The effects of the significant LD block resulted in significant differences in the carcass traits, as shown in Supplementary Figures 2, 3. The lowest and highest phenotypic values belonged to homozygotes, whereas heterozygotes had intermediate values. Broilers with homozygous mutations of the LD block on GGA24 (GGA24: 5.73–5.75 Mb) had higher carcass weights and higher FIs than those with homozygous wild type. Broilers with homozygous mutations of the LD block on GGA3 (GGA3: 53.03–53.08 Mb) had lower WThP, ThP, and ThMP than those with homozygous wild type.

### Bayesian Analysis

**FIGURE 4:**
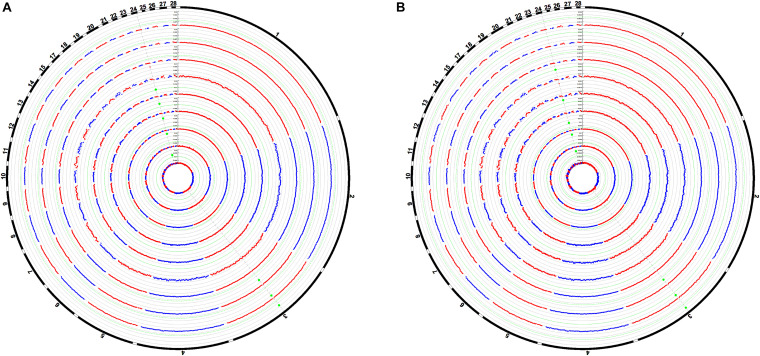
Manhattan plots of the average proportion of genetic variance **(A)** and phenotypic variance **(B)** explained by the 1-Mb window for all traits. Each dot represents a 1-Mb window. The circle Manhattan plots from the inside to the outside are BW42, CW, EW, WThW, ThW, ThMW, WThP, ThP, and ThMP. The dotted green lines indicate the 1.00% threshold of genetic variance and the 0.30% threshold of the phenotypic variance explained by the windows.

The Manhattan plots of the genetic variance and phenotypic variance explained by each 1-Mb window on different chromosomes for all traits are shown in Figures 4A,B, respectively. The genetic variance and phenotypic variance explained by the top 1-Mb windows for all traits are listed in Table 3. Among a total of 2014 windows, the top window explained 0.95–4.41% of the genetic variance and 0.29–1.13% of the phenotypic variance of the weight traits. For the carcass composition traits, the top window explained 2.00–3.00% of the genetic variance and 0.39–0.45% of the phenotypic variance. The top windows for all traits overlapped with the significant regions obtained from the GWAS.

**TABLE 3:** genetic variance and phenotypic variance explained by top 1-Mb windows for all traits using Bayes C_π_.

**Top window^1^**	**Traits^2^**	**GVE (%)^3^**	**PVE (%)^4^**
Chr 24 (5.05–5.97 Mb)	BW42	1.31	0.47
	CW	2.92	0.91
	EW	4.41	1.13
	WThW	1.43	0.39
	ThW	1.08	0.29
	ThMW	0.95	0.31
Chr 3 (53.02–54.00 Mb)	WThP	2.51	0.40
	ThP	3.00	0.39
	ThMP	2.00	0.45

*^1^Window position in galGal6. ^2^Body weight at 42 days of age (BW42), carcass weight (CW), eviscerated weight (EW), whole thigh weight (WThW), thigh weight (ThW), thigh muscle weight (ThMW), whole thigh percentage (WThP), thigh percentage (ThP), thigh muscle percentage (ThMP), and feed intake from 28 to 42 days of age (FI). ^3^Average proportion of genetic variance explained by 1-Mb window. ^4^Average proportion of phenotypic variance explained by 1-Mb window.*

### Estimation of SNP Effect Size

**FIGURE 5:**
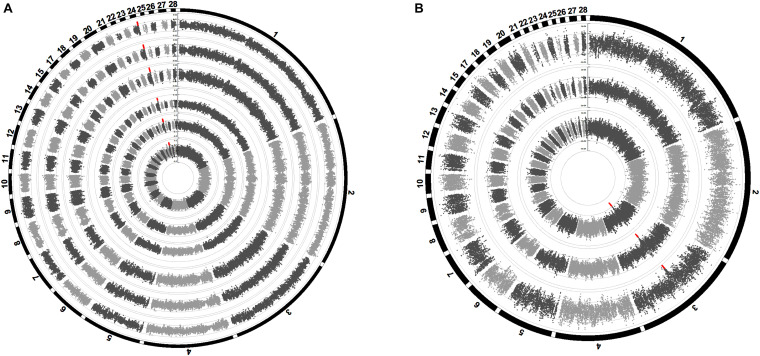
Manhattan plots of SNP effect sizes for the carcass traits **(A)** and carcass composition traits **(B)**. Each dot represents an SNP. The SNP effect sizes are shown on the *y*-axis. The circle Manhattan plots from the inside to the outside are BW42, CW, EW, WThW, ThW, ThMW, WThP, ThP, and ThMP. The SNPs highlighted in red are suggestively significant in GWAS.

The Manhattan plots of the SNP effect size are shown in Figure 5. The SNPs highlighted in red were suggestively significant in GWAS. The SNP effect size of the significant SNPs in GWAS of the six weight traits was between 0.008 and 0.155. The SNP effect size of the significant SNPs in GWAS of the three carcass composition traits was between −3.01e−4 and −2.06e−4. The SNP effect size of the significant SNPs in GWAS of the weight traits and carcass composition traits reached the top 0.24 and 0.26% of the whole-genome SNPs, respectively.

### Phylogenetic Analysis

**FIGURE 6:**
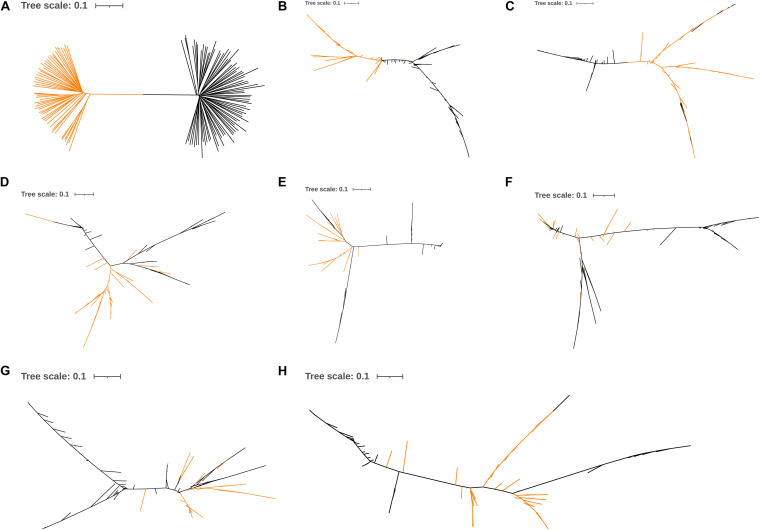
Neighbor-joining tree based on the whole-genome SNPs **(A)**, candidate genes **(B,C)**, and 5 random 100-Kb regions **(D–H)**. The yellow line represents the Chahua chickens and Daweishan mini chickens, and the black line represents Line B.

The phylogenetic analysis showed that the Line B chickens were separated from the Chahua and Daweishan mini chickens based on whole-genome SNPs, the SNPs within *DRD2*, or the SNPs within *ADGRG6*, respectively (Figures 6A–C). In contrast, the Line B chickens were not separated from the Chahua and Daweishan mini chickens when five random-selected 100 Kb regions were used (Figures 6D–H).

### Selective Sweep Analysis

**FIGURE 7:**
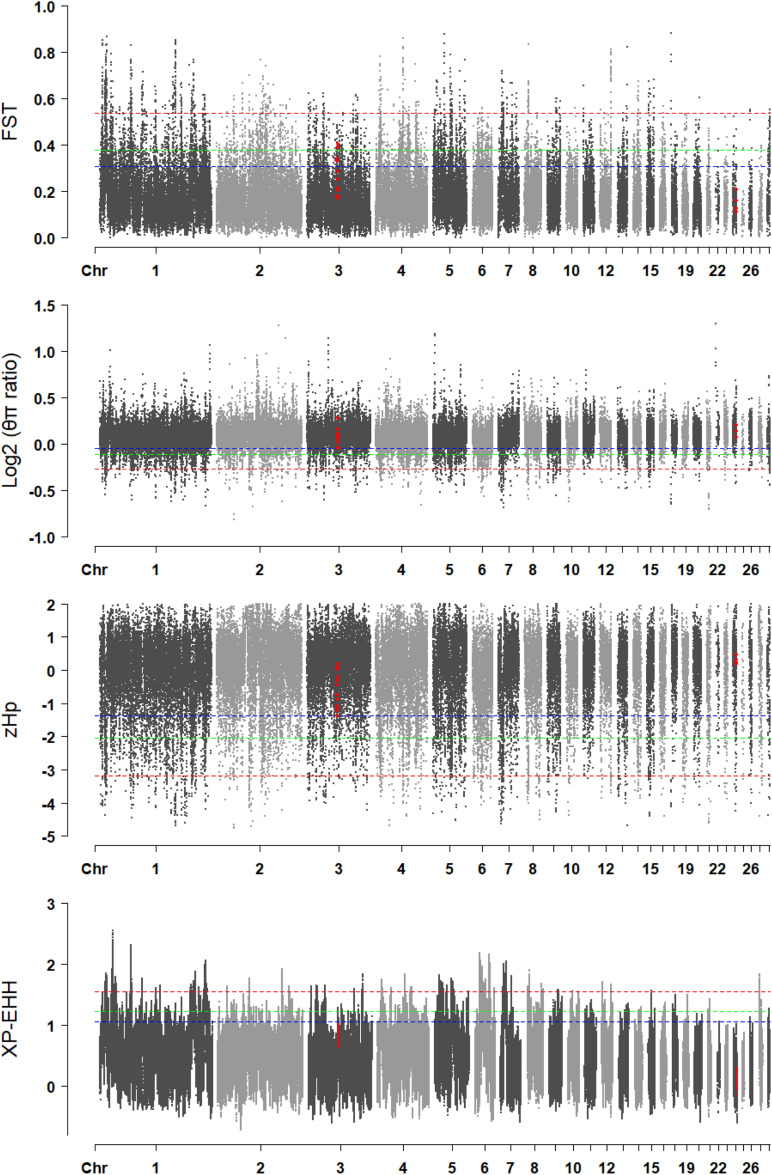
The visualization of the selective sweep analysis. The horizontal blue, green, and red lines indicate the thresholds for 10% genome-wide significance, 5% genome-wide significance, and 1% genome-wide significance, respectively.

A genome-wide selective sweep analysis was performed with the fast-growing Line B chickens, the Chahua chickens, and the Daweishan mini chickens (Figure 7). The *F*_*ST*_ values in *DRD2* were between 0.11 and 0.21, and the *F*_*ST*_ values in *ADGRG6* were between 0.17 and 0.40. Only the values in *ADGRG6* reached the top 5% threshold (0.38) of the whole genome. The *log*_2_⁡(π*ratio*) values in *DRD2* were between 0.07 and 0.20, and those in *ADGRG6* were between -0.04 and 0.27, reaching the top 10% threshold (-0.04) of the whole genome. The zHp values in *DRD2* were between 0.18 and 0.45, and those in *ADGRG6* were between -1.37 and 0.19, reaching the top 10% threshold (-1.37) of the whole genome. The XP-EHH values in *DRD2* were between -0.12 and 0.30, and those in *ADGRG6* were between 0.64 and 1.02. Overall, only the *ADGRG6* region showed some degree of genetic and nucleotide differentiation between large and small breeds.

## Discussion

Genotype imputation has been widely used in GWAS to boost power (Spencer et al., 2009). This method can aid in identifying many novel SNPs and QTLs associated with phenotypes of interest. In previous GWAS, imputation from low-density SNP chip genotypes to the WGS level was implemented in chickens (Huang et al., 2018; Li D. et al., 2020), pigs (Yan et al., 2018), and cattle (Höglund et al., 2015). Imputed genotypes with sufficiently high imputation accuracy are necessary to obtain reliable results in follow-up analyses, such as GWAS. Ni et al. (2017) reported that the post-imputation filtering criterion should be 0.80 to ensure the high accuracy of the imputed WGS data. In the current study, the average allelic *R*^2^ value and the genotype concordance rate between the imputed and true genotypes were 0.89 and 0.91, respectively.

The heritability estimates for the six weight traits were moderate to high (0.30–0.39), showing high consistency with previous reports (Demeure et al., 2013). However, the estimates were lower than 0.56 in medium-growing broilers at 44 days of age (Xu et al., 2016). Our heritability estimate for ThP was 0.22, which was slightly lower than 0.37, as reported by Demeure et al. (2013). The likely reason is the genetic background difference in the chicken lines.

A 24.08 Kb QTL on GGA24 (GGA24: 5.73–5.75 Mb) was identified for the six weight traits. According to the Animal QTL Database^8^, this region has been recorded as a QTL (GGA24: 4.3–6.0 Mb) for BW08 in an F2 population (Taiwan local chicken line L2 × experimental Rhode Island Red line; Lien et al., 2017). This region contains only one candidate gene (*DRD2*), which encodes the D2 subtype of the dopamine receptor. The gene is highly expressed in the basal ganglia (Missale et al., 1998), which is a control center for movement. Neurotransmission mediated by *DRD2* is known to have a key role in the control of movement. *DRD2* TaqIA polymorphisms were correlated with body mass in previous studies (Spitz et al., 2000; Thomas et al., 2001). The density of the *DRD2* receptors can affect food reinforcement to influence energy intake (Epstein et al., 2007). Down-regulation of *DRD2* receptors leads to increased food intake and weight gain (Epstein et al., 2007). In the current study, significant differences in weight traits and feed intake were observed between birds with different genotypes. The mRNA expression of *DRD2* in the thigh muscle was not detected by quantitative polymerase chain reaction (Q-PCR; data not shown). The Galbase data shows that *DRD2* has high mRNA expression in chickens’ brain tissue^9^. Therefore, brain tissue should be obtained and tested in a future study.

We focused specifically on the thigh traits in the carcass composition traits in the current study. A 42.52 kb genomic region on GGA3 (GGA3: 53.03–53.08 Mb) was identified for WThP and ThP. This QTL was not reported previously. This region contained only one candidate gene (*ADGRG6*), which encodes the G-protein-coupled receptor 126. Ravenscroft et al. (2015) proved that *ADGRG6* is critical for myelination of peripheral nerves in humans and mutations of *ADGRG6* are responsible for severe arthrogryposis multiplex congenita. Soranzo et al. (2009) found that mutations in *ADGRG6* are related to trunk length, hip axis length, and height. Numerous studies have shown that *ADGRG6* is associated with adult height and pediatric stature (Hirschhorn et al., 2001; Xu et al., 2002; Gudbjartsson et al., 2008; Lettre et al., 2008; Sovio et al., 2009; Liu et al., 2010; Zhao et al., 2010). Kou et al. (2013) found that *ADGRG6* was highly expressed in cartilage, and the knockdown of *ADGRG6* in zebrafish caused delayed ossification of the developing spine. It is believed that *ADGRG6* may affect both adolescent idiopathic scoliosis susceptibility and height through abnormal spinal development and/or growth. Karner et al. (2015) also proved that the loss of *ADGRG6* in osteochondroprogenitor cells alters cartilage biology and spinal column development.

Regarding weight traits, the significant region of BW42, CW, and EW detected by multivariate GWAS was consistent with that detected by univariate GWAS. The significance levels of BW42, CW, EW, WThW, ThW, and ThMW were lower in the multivariate GWAS than the univariate GWAS. Therefore, it was believed that the significant region on the GGA24 had a greater impact on the overall body weight of the chicken than on the thigh weight. Regarding carcass composition traits, the significant region of WThP and ThP detected by multivariate GWAS was consistent with that detected by univariate GWAS. Therefore, it was believed that the significant region on the GGA3 had an impact on WThP, ThP, and ThMP.

It was interesting that significant differences in thigh composition traits were observed between birds with different genotypes of the associated variants. The flavor of thigh meat is usually better than that of the breast muscle because its intramuscular fat content is three times higher than that of the latter (Zhao et al., 2007; Fu et al., 2014). The significant SNPs could be used in genomic selection programs to improve the percentage of thigh meat (Zhang et al., 2015).

In the current study, the results of the Bayesian analysis and the estimation of the SNP effect size verified the GWAS results. The SNP effect sizes of the significant SNPs in GWAS of the weight and carcass composition traits reached the top 0.24 and 0.26% of the whole genome SNPs, respectively. Among all traits, the top 1-Mb sliding windows, which overlapped with the significant regions in GWAS, explained 0.95–4.41% of genetic variation and 0.29–1.13% of phenotypic variation. In contrast, the remaining 1-Mb sliding window explained no more than 0.24% of genetic variation and no more than 0.08% of phenotypic variation.

In the study by Yoshida et al. (2017), the genes located in the top ten 1-Mb windows were identified as strong functional candidate genes, and the top window for the growth traits explained 3.71 and 3.61% of genetic variance, respectively. In a study of a pure line of broilers, the window with the largest effect for the bodyweight, breast meat, and leg score explained 2.5, 1.14, and 1.12% of the genetic variation, respectively (Fragomeni Bde et al., 2014).

In the current study, the phylogenetic tree indicated genetic differentiation in *DRD2* and *ADGRG6* between the Line B chickens and the small-sized breeds because the former could not be completely separated from the Chahua and Daweishan mini chickens when five random-selected 100 Kb regions were used. In the selective sweep analysis of chickens, windows with thresholds of 1 or 5% outliers were typically identified as candidate regions (Yin et al., 2019; Li W. et al., 2020; Luo et al., 2020; Weng et al., 2020). The *F*_*ST*_ values in *ADGRG6* reached the top 5% threshold, whereas the *log*_2_⁡(π*ratio*) values and zHp values reached the top 10% threshold of the whole genome. *ADGRG6* showed some degree of genetic and nucleotide differentiation between the fast-growing Line B chickens and the Chahua and Daweishan mini chickens. However, the *DRD2* showed no related signals. Since the significant SNPs were located in the introns and the upstream and intergenic regions of the candidate genes, it is challenging to investigate causative mutations, which will be performed in a future study.

## Conclusion

In summary, the genomic heritability estimates of nine chicken carcass traits ranged from moderate to high (0.21 to 0.39). Twelve genome-wide significant SNPs and 118 suggestively significant SNPs were detected. One 24.08 Kb region (GGA24: 5.73–5.75 Mb) for six weight traits and one 42.52 Kb region (GGA3: 53.03–53.08 Mb) for three thigh-related carcass traits were identified. The significant SNPs could be used in genomic selection programs to improve the weight traits and thigh composition traits. In the QTL regions, *DRD2* was the only major-effect candidate gene for weight traits, and *ADGRG6* was the only major-effect candidate gene for carcass composition traits. Some degree of genetic differentiation in *ADGRG6* between large-sized and small-sized breeds was observed. Our results supply essential information for causative mutation identification of carcass traits in broilers.

## Data Availability Statement

The datasets presented in this study can be found in online repositories. The names of the repository and accession numbers can be found below: https://bigd.big.ac.cn/gsa, CRA002454 and CRA004023.

## Ethics Statement

All experimental procedures with broilers were performed according to the Guidelines for Experimental Animals established by the Ministry of Science and Technology (Beijing, China). Ethical approval on animal survival was given by the animal welfare and ethics committee of the Institute of Animal Sciences (IAS) and the Chinese Academy of Agricultural Sciences (CAAS, Beijing, China) with the following reference number: IAS2019–44.

## Author Contributions

XY contributed to data collection, data analysis and interpretation, and wrote the manuscript. JS contributed to data collection and manuscript revision. GZ, MZ, and JW designed the research and revised manuscript. WL, XT, FF, and DL contributed to the chicken raising, sampling, and data collection. RL designed the research and contributed to data collection, data analysis and interpretation, and wrote the manuscript. All authors submitted comments on the draft and approved the final manuscript.

## Conflict of Interest

FF and DL were employed by the Foshan Gaoming Xinguang Agricultural and Animal Industrials Corporation. The remaining authors declare that the research was conducted in the absence of any commercial or financial relationships that could be construed as a potential conflict of interest.

## Footnotes

1 http://www.fao.org/home/en/

2 http://broadinstitute.github.io/picard/

3 https://github.com/genetics-statistics/GEMMA/releases

4 https://cran.r-project.org/package=CMplot

5 http://genome-asia.ucsc.edu/cgi-bin/hgGateway?hgsid=472768848_otkBtCHKhHMTV1xrxHuq737iivJ1

6 https://github.com/YinLiLin/hibayes

7 https://www.dissect.ed.ac.uk

8 https://www.animalgenome.org/cgi-bin/QTLdb/GG/index

9 http://animal.nwsuaf.edu.cn/code/index.php/ChickenVar
