# Supplementary material for: Identification of Major Loci and Candidate Genes for Meat Production-Related Traits in Broilers
Source: Front Genet. 2021 Mar 30;12:645107. doi: 10.3389/fgene.2021.645107 (PMC8042277; doi:10.3389/fgene.2021.645107)
Supplement: Supplementary file 1 [file Table_1.DOCX]

**TABLE S1 Distribution of SNPs used in the GWAS after post-imputation filtering**

| **GGA^1^** | **Physical Map, Mb^2^** | **No. of SNP markers** | **Average distance, kb** | **allelic R2** | **genotype concordance rate** |
| --- | --- | --- | --- | --- | --- |
| 1 | 197.61 | 1,475,905 | 0.134 | 0.901 | 0.907 |
| 2 | 149.68 | 1,112,282 | 0.135 | 0.894 | 0.912 |
| 3 | 110.84 | 849,331 | 0.131 | 0.894 | 0.916 |
| 4 | 91.32 | 697,742 | 0.131 | 0.896 | 0.902 |
| 5 | 59.81 | 395,240 | 0.151 | 0.890 | 0.907 |
| 6 | 36.37 | 296,262 | 0.123 | 0.887 | 0.913 |
| 7 | 36.74 | 272,657 | 0.135 | 0.891 | 0.920 |
| 8 | 30.22 | 188,666 | 0.160 | 0.901 | 0.912 |
| 9 | 24.15 | 169,847 | 0.142 | 0.895 | 0.923 |
| 10 | 21.12 | 161,321 | 0.131 | 0.901 | 0.921 |
| 11 | 20.20 | 148,634 | 0.136 | 0.910 | 0.899 |
| 12 | 20.39 | 182,434 | 0.112 | 0.905 | 0.920 |
| 13 | 19.17 | 121,419 | 0.158 | 0.884 | 0.921 |
| 14 | 16.22 | 99,624 | 0.163 | 0.896 | 0.921 |
| 15 | 13.06 | 82,644 | 0.158 | 0.889 | 0.918 |
| 17 | 10.76 | 60,465 | 0.178 | 0.886 | 0.948 |
| 18 | 11.37 | 76,632 | 0.148 | 0.891 | 0.933 |
| 19 | 10.32 | 62,640 | 0.165 | 0.885 | 0.937 |
| 20 | 13.90 | 98,879 | 0.141 | 0.892 | 0.914 |
| 21 | 6.84 | 36,010 | 0.190 | 0.893 | 0.897 |
| 22 | 5.46 | 8,839 | 0.618 | 0.885 | 0.885 |
| 23 | 6.15 | 18,803 | 0.327 | 0.861 | 0.862 |
| 24 | 6.49 | 32,992 | 0.197 | 0.885 | 0.926 |
| 25 | 3.98 | 937 | 4.248 | 0.855 | 0.827 |
| 26 | 6.06 | 19,203 | 0.316 | 0.868 | 0.901 |
| 27 | 8.08 | 13,136 | 0.615 | 0.868 | 0.855 |
| 28 | 5.12 | 19,985 | 0.256 | 0.893 | 0.920 |

^1^ *Gallus gallus* chromosome.

^2^ Physical length of the chromosome based on Gallus gallus-6.0.
