# Supplementary material for: Identification of Major Loci and Candidate Genes for Meat Production-Related Traits in Broilers
Source: Front Genet. 2021 Mar 30;12:645107. doi: 10.3389/fgene.2021.645107 (PMC8042277; doi:10.3389/fgene.2021.645107)
Supplement: Supplementary file 2 [file Table_2.DOCX]

**TABLE S2 Estimates of heritabilities (diagonal), phenotypic correlations (below diagonal), genetic correlations (above diagonal) and their standard errors among carcass traits.**

|  | **BW42** | **CW** | **EW** | **WThW** | **ThW** | **ThMW** | **WThP** | **ThP** | **ThMP** |
| --- | --- | --- | --- | --- | --- | --- | --- | --- | --- |
| **BW42** | **0.39±0.08** | 0.99±0.009* | 0.93±0.04* | 0.89±0.04* | 0.88±0.05* | 0.79±0.07* | -0.18±0.21 | -0.11±0.21 | -0.05±0.18 |
| **CW** | 0.95±0.004 | **0.36±0.08** | 0.92±0.04* | 0.86±0.05* | 0.84±0.06* | 0.78±0.07* | -0.26±0.21 | -0.17±0.22 | -0.07±0.19 |
| **EW** | 0.79±0.01 | 0.80±0.01 | **0.30±0.08** | 0.90±0.06* | 0.88±0.07* | 0.81±0.08* | -0.03±0.23 | 0.02±0.23 | 0.08±0.20 |
| **WThW** | 0.83±0.01 | 0.85±0.01 | 0.73±0.01 | **0.32±0.08** | 0.98±0.004* | 0.93±0.03* | 0.27±0.20 | 0.31±0.20 | 0.29±0.17 |
| **ThW** | 0.81±0.01 | 0.83±0.01 | 0.71±0.01 | 0.99±0.0004 | **0.31±0.08** | 0.96±0.02* | 0.28±0.21 | 0.38±0.19 | 0.38±0.17* |
| **ThMW** | 0.78±0.01 | 0.81±0.01 | 0.71±0.02 | 0.93±0.004 | 0.94±0.003 | **0.34±0.08** | 0.32±0.19 | 0.45±0.18* | 0.56±0.12* |
| **WThP** | -0.10±0.03 | 0.02±0.03 | 0.04±0.03 | 0.47±0.02 | 0.50±0.02 | 0.43±0.02 | **0.21±0.07** | 0.94±0.02* | 0.76±0.09* |
| **ThP** | -0.04±0.03 | 0.07±0.03 | 0.08±0.03 | 0.52±0.02 | 0.56±0.02 | 0.49±0.02 | 0.98±0.001 | **0.22±0.07** | 0.91±0.05* |
| **ThMP** | -0.01±0.03 | 0.09±0.03 | 0.12±0.03 | 0.44±0.02 | 0.48±0.02 | 0.61±0.02 | 0.81±0.01 | 0.83±0.01 | **0.33±0.08** |

Significant LRT values are marked with an asterisk.

Body weight at 42 days of age (BW42), carcass weight (CW), eviscerated weight (EW), whole thigh weight (WThW), thigh weight (ThW), thigh muscle weight (ThMW), whole thigh percentage (WThP), thigh percentage (ThP), thigh muscle percentage (ThMP), feed intake from 28 to 42 days of age (FI).
