# Supplementary material for: Identification of Major Loci and Candidate Genes for Meat Production-Related Traits in Broilers
Source: Front Genet. 2021 Mar 30;12:645107. doi: 10.3389/fgene.2021.645107 (PMC8042277; doi:10.3389/fgene.2021.645107)
Supplement: Supplementary file 3 [file Presentation_1.PPTX]

## Slide 1
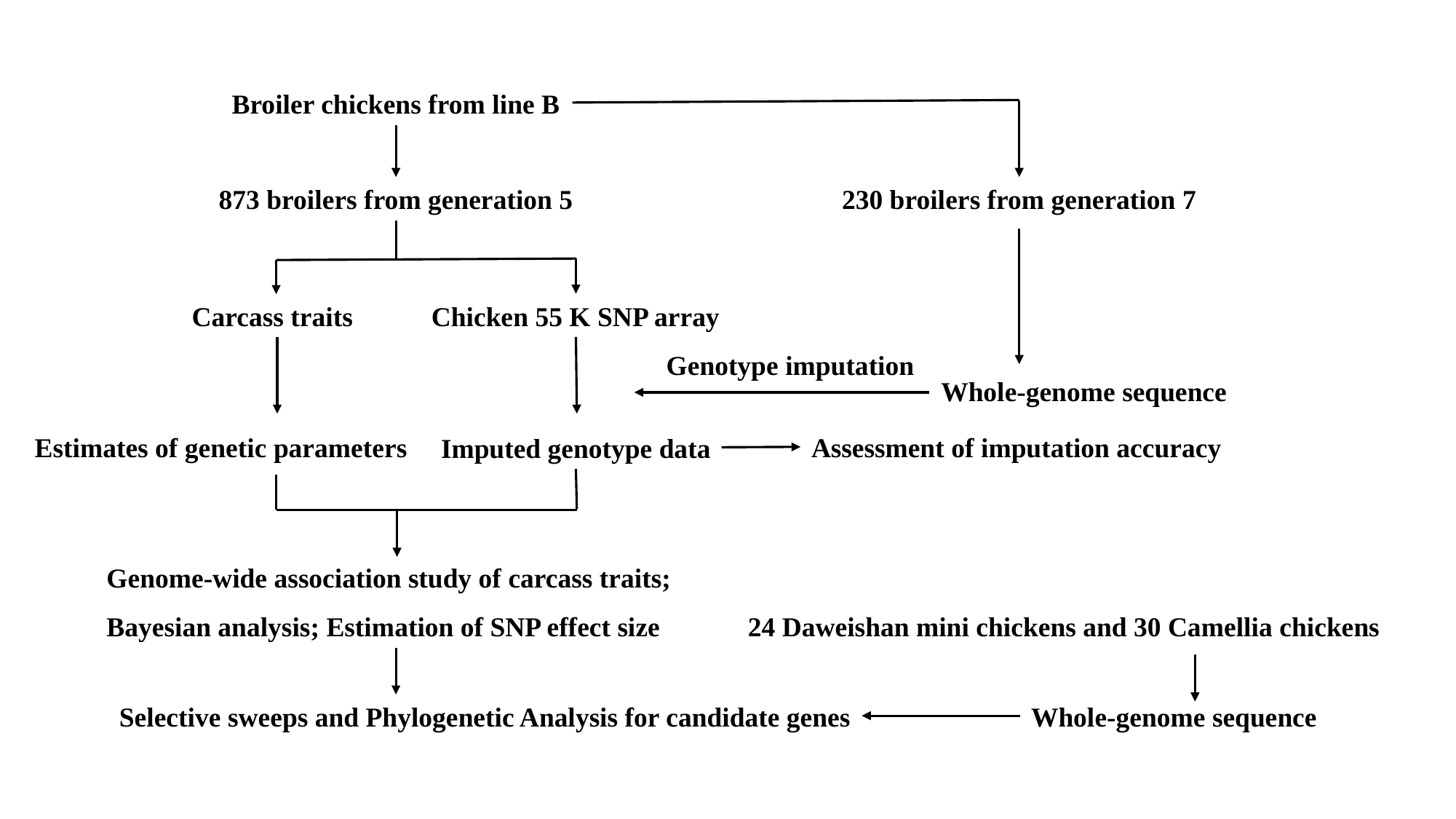

Broiler chickens from line B
873 broilers from generation 5
230 broilers from generation 7
Chicken 55 K SNP array
Carcass traits
Genotype imputation
Whole-genome sequence
Estimates of genetic parameters
Assessment of imputation accuracy
Imputed genotype data
Genome-wide association study of carcass traits; Bayesian analysis; Estimation of SNP effect size
24 Daweishan mini chickens and 30 Camellia chickens
Selective sweeps and Phylogenetic Analysis for candidate genes
Whole-genome sequence
