# Supplementary figures and images for: Identification of Major Loci and Candidate Genes for Meat Production-Related Traits in Broilers
Source: Front Genet. 2021 Mar 30;12:645107. doi: 10.3389/fgene.2021.645107 (PMC8042277; doi:10.3389/fgene.2021.645107)

## Slide 1
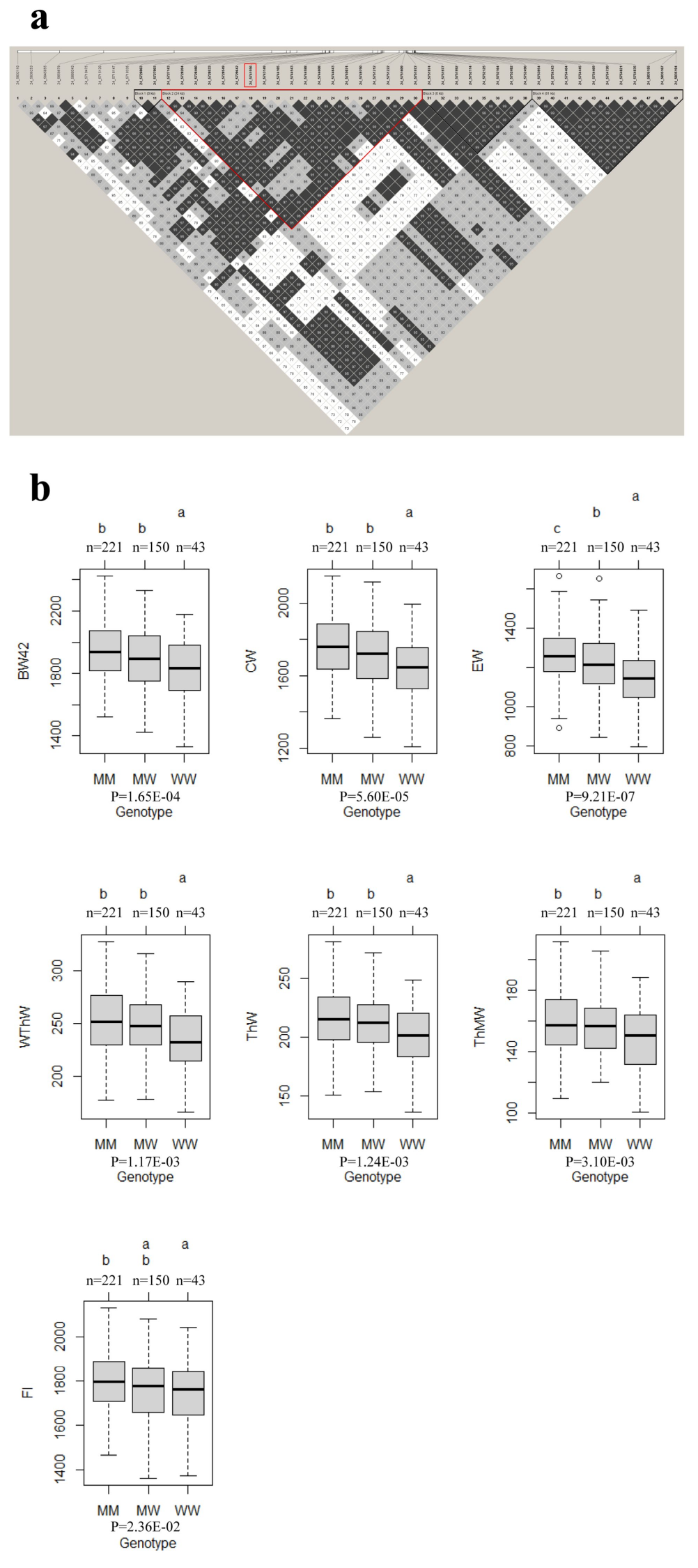

Supplement: Supplementary file 4 [file Presentation_2.PPTX]

## Slide 1
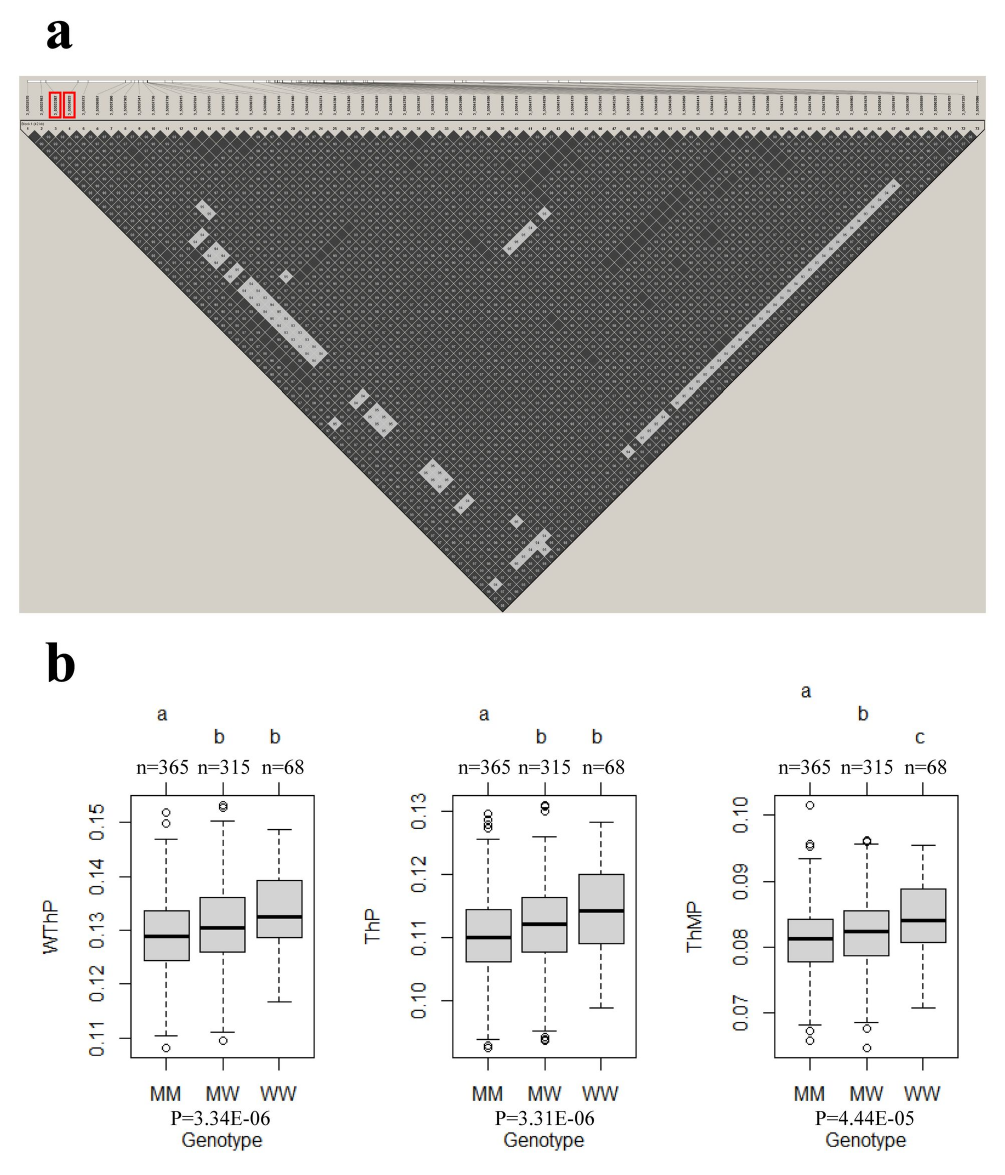

Supplement: Supplementary file 5 [file Presentation_3.PPTX]
